# Health and socio-demographic profile of women of reproductive age in rural communities of southern Mozambique

**DOI:** 10.1371/journal.pone.0184249

**Published:** 2018-02-02

**Authors:** Charfudin Sacoor, Beth Payne, Orvalho Augusto, Faustino Vilanculo, Ariel Nhacolo, Marianne Vidler, Prestige Tatenda Makanga, Khátia Munguambe, Tang Lee, Eusébio Macete, Peter von Dadelszen, Esperança Sevene, the CLIP Working Group

**Affiliations:** 1 Centro de Investigação em Saúde da Manhiça (CISM), Manhiça, Mozambique; 2 University of British Columbia (UBC), Vancouver, Canada; 3 Simon Fraser University (SFU), Burnaby, Canada; 4 Midlands State University (MSU), Gweru, Zimbabwe; 5 Faculdade de Medicina, Universidade Eduardo Mondlane, Maputo, Mozambique; 6 Direcção Nacional de Saúde, Ministério da Saúde, Maputo, Mozambique; 7 Department of Obstetrics and Gynaecology, King’s College, London, United Kingdom; Stellenbosch University, SOUTH AFRICA

## Abstract

Reliable statistics on maternal morbidity and mortality are scarce in low and middle-income countries, especially in rural areas. This is the case in Mozambique where many births happen at home. Furthermore, a sizeable number of facility births have inadequate registration. Such information is crucial for developing effective national and global health policies for maternal and child health. The aim of this study was to generate reliable baseline socio-demographic information on women of reproductive age as well as to establish a demographic surveillance platform to support the planning and implementation of the Community Level Intervention for Pre-eclampsia (CLIP) study, a cluster randomized controlled trial. This study represents a census of all women of reproductive age (12–49 years) in twelve rural communities in Maputo and Gaza provinces of Mozambique. The data were collected through electronic forms implemented in Open Data Kit (ODK) (an app for android based tablets) and household and individual characteristics. Verbal autopsies were conducted on all reported maternal deaths to determine the underlying cause of death. Between March and October 2014, 50,493 households and 80,483 women of reproductive age (mean age 26.9 years) were surveyed. A total of 14,617 pregnancies were reported in the twelve months prior to the census, resulting in 9,029 completed pregnancies. Of completed pregnancies, 8,796 resulted in live births, 466 resulted in stillbirths and 288 resulted in miscarriages. The remaining pregnancies had not yet been completed during the time of the survey (5,588 pregnancies). The age specific fertility indicates that highest rate (188 live births per 1,000 women) occurs in the age 20–24 years old. The estimated stillbirth rate was 50.3/1,000 live and stillbirths; neonatal mortality rate was 13.3/1,000 live births and maternal mortality ratio was 204.6/100,000 live births. The most common direct cause of maternal death was eclampsia and tuberculosis was the most common indirect cause of death. This study found that fertility rate is high at age 20–24 years old. Pregnancy in the advanced age (>35 years of age) in this study was associated with higher poor outcomes such as miscarriage and stillbirth. The study also found high stillbirth rate indicating a need for increased attention to maternal health in southern Mozambique. Tuberculosis and HIV/AIDS are prominent indirect causes of maternal death, while eclampsia represents the number one direct obstetric cause of maternal deaths in these communities. Additional efforts to promote safe motherhood and improve child survival are crucial in these communities.

## Introduction

Reliable statistics on maternal and perinatal morbidity and mortality are scarce in low and middle-income countries (LMIC), especially in rural areas. This information is crucial for developing effective national and global health policies for maternal and child health. In many African countries, it is common for people to be born, grow and die without being formally registered. In Africa, only Mauritius and the Seychelles have complete registration of births [1], deaths and causes of death. Fifty-seven million deaths occurred globally in 2013, approximately 38 million (two-thirds) were not formally registered [2]. Low and middle-income countries account for 99% (284,000) of all maternal deaths, and Sub-Saharan Africa accounts for 56% (162,000) of these deaths [3]. The cause of death is often unknown and as a result policy makers and public health practitioners are not well equipped to appropriately define priorities, design effective public health strategies or accurately measure the impact of programmes. In response to these challenges, there are current initiatives in place for data collection in low-income countries, but most of these are confined to small, or restricted, study areas. The INDEPTH Network platform (http://www.indepth-network.org) is an example of such an initiative. In Mozambique, there are two surveillance site members of this network, namely the Manhiça HDSS and the Chokwé HDSS, established to monitor health and population statistics and to facilitate biomedical research. The Manhiça HDSS has been running since 1996 and has provided reliable data to guide health policies for disease control [4].

In Mozambique, reliable data on maternal morbidity and mortality is a challenge. The available health facility data largely reflects the situation in urban areas and among social groups with access to health services [5, 6]. National figures often rely on estimates extrapolated to the whole country, thus not likely to represent all regions and information on cause of death is always not available [7–9]. Maternal mortality ratio (MMR) in the country was estimated in 2011 at 408 per 100,000 live births [10], one of the highest in the world. In places where data is available, the hypertensive disorders of pregnancy (HDP) rank as a top cause of maternal death, responsible for 18% of maternal deaths [11]. Annually 62,000 to 77,000 women and 500,000 infants die as a result of the HDPs worldwide [11]. In Mozambique, the HDPs are estimated to be the third leading cause of maternal mortality [7–9]. In an effort to reduce maternal and perinatal deaths associated with pre-eclampsia in LMIC, the Community Level Interventions in Pre-eclampsia (CLIP) trial was initiated in Mozambique, Nigeria, India and Pakistan (clinical trial registration number: NCT01911494). This is a cluster randomized controlled trial to test the impact of task-sharing the initial diagnosis and management of the HDPs to community-based health workers.

The most recent community-based demographic health survey reported in 2013 in the country, did not include a sufficient sample size to describe characteristics at the district or sub-district level [10]. Thus, as part of baseline assessment of the CLIP trial, a census of all women of reproductive age (WRA) (12–49 years) in 12 rural communities in northern Maputo Province and southern Gaza Province was conducted. The aims of this household census were to enumerate all WRA, generate reliable socio-demographic information, as well as to establish a demographic surveillance platform to support the planning and the implementation of the upcoming clinical trial. In this paper, we report the results of the household census.

## Methods

### Study design and setting

**Fig 1:**
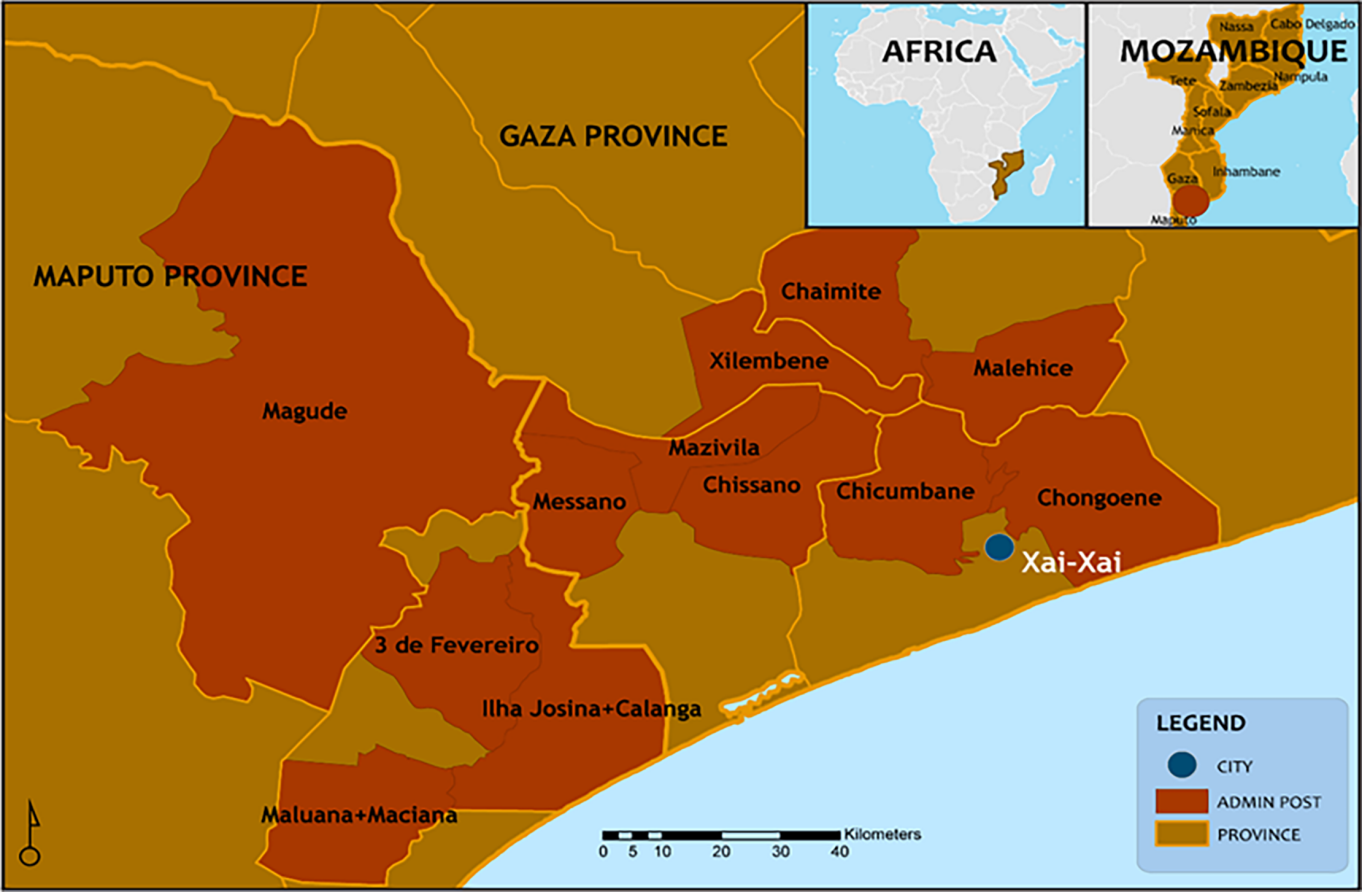
Geographical location of study areas (clusters) in Maputo and Gaza provinces.

The census was conducted in 2014 to identify the socio-demographic information of the population: number and membership of each household, names, ages, reproductive history, and mortality. The census included all women of reproductive age in 12 rural areas in two provinces in southern Mozambique, namely Maputo and Gaza. The methodology used was based on the process in use by the Health Demographic and Surveillance System (HDSS), this facilitated follow-up all women surveyed [4, 12]. Each of the twelve rural areas was defined by a geographic region, which contained a minimum population size of 25,000 inhabitants. This number was defined as the minimum total population that would result in at least one maternal death per year as per the data from the 2007 national census of Mozambique [13]. The national administrative structure used to delimit each region was the Administrative Posts (AP) that represents the 3rd administrative level in Mozambique after the Province and Districts; in cases where the AP had insufficient population, an additional AP was added and in situations where the AP had more than 25,000 inhabitants a delimitation of portions of the AP was done by excluding some localities or neighbourhoods. As census data, will be used in the CLIP cluster randomized control trial, the selection of study regions aimed to avoid contamination between regions. To avoid contamination, no areas were geographically contiguous; all regions were separated by non-inhabited areas such as wide bush and forest, swampy areas, rivers and areas of inundation. The data used to define the 12 clusters were based on statistics from the National Census of 2007 carried out by National Institute of Statistics of Mozambique [13]. As shown in Fig 1, in Maputo Province there were four clusters namely: Maluana & Maciana, Calanga & Ilha Josina, 3 de Fevereiro and Magude while in Gaza there were eight clusters: Messano, Mazivila, Xilembene, Chissano, Chicumbane, Chongoene, Malehice and Chaimite.

### Inclusion and exclusion criteria

All households with one or more woman of reproductive age (12 to 49 years) in the selected clusters were included in the census. Further, in order to be included in the census, the women must have lived in the household for more than 30 days prior to the date of the census and had the intention to live in this household as permanent resident for at least six months following the census. In addition, women of reproductive age who died in the 12 months preceding the survey were included to provide mortality data on this group.

### Data collection tools

The data were collected using android tablets equipped with Open Data Kit (ODK https://opendatakit.org) suited with ODK Collect version 1.4. The data collection forms contained questions on household characteristics, socio-demographic characteristics, obstetric and medical histories.

Before implementation, the electronic forms were tested by members of the IT team as well as piloted in the field. Data was uploaded to a central server from tablets on a weekly basis.

Household information included questions regarding the materials used in construction, the number of buildings, the presence of a latrine and kitchen, the source of drinking water, the use of electricity and the main type of fuel for cooking. WRA were asked their name, date of birth, relationship with the head of household, level of education, occupation, religion, marital status.

To determine the causes of death, a verbal autopsy (VA) was conducted for all deaths among WRA. The VA required an interview with family members or caregivers about the circumstances of the death. Verbal autopsies are regularly used in areas where routine death registration is non-existent or inadequate [14]. The 2012 WHO VA tool was translated to Portuguese and customized for electronic use with ODK for this study.

### Data collection procedures

All the field workers were local residents; both male and female, most had a minimum education level of grade 12. In areas with few grade 12 candidates, data collectors were recruited with as little as 10th grade completed. The enumeration of households included painting household IDs on the door; this was done by 38 field workers. These IDs represent the locality and neighbourhood of the household. In addition to the 38 field officers mentioned above, 208 interviewers were recruited. All electronic data was returned to the central office at Centro de Investigação em Saúde da Manhiça (CISM) for upload onto the server every Friday. A few select sites in Gaza used a remote 3G secure connection to upload the data. After each upload, all the data were removed from the tablets to ensure patient confidentiality.

To determine the cause of maternal deaths verbal autopsy and physician review was used. Three independent physicians reviewed each verbal autopsy using the International Classification of Diseases (ICD-10) to assign a maximum of two diagnoses responsible for the death. The cause of death was determined based on agreement of diagnoses of physicians.

### Statistical analysis

Descriptive analyses were performed using Stata13 package (version 13.1, Stata Corp, Texas, US). No inferential statistics (p-values or 95%CI) is presented because the data came from a census, therefore the population parameters are directly calculated. The indicators presented were based on internationally accepted definitions. The age-specific fertility rate (ASFR) was defined as the number of live births per 1,000 WRA in a specific age interval. Stillbirth rate (SBR) was defined as the number of foetal deaths after 28 weeks divided by total live births plus stillbirths per thousands. Neonatal mortality ratio (NMR) was defined as infant deaths under 28 days divided by total live births. The maternal mortality ratio (MMR) was defined as all deaths of women as a result of pregnancy, childbirth and post-partum up to 42 days divided by total live births. Finally, the death rate for WRA was derived from all deaths of women of reproductive age (12–49 years) per 1,000 population[15, 16].

The first component of the Principal component analysis (PCA) was used to generate the poverty index from household characteristics: type of wall, type of latrine, access to transport, source of drinking water, electricity in the household and ownership of numerous household items (TV, radio, bed, clock or watch, and iron) among other. This index was categorized into quintiles of poverty [17, 18].

To mitigate recall bias of the number of children reported we also asked about children not leaving with the mother and the family. We did consistency checks of the total pregnancies and pregnancies outcomes in the last 12 months prior to the census per woman. Women who reported more than 3 pregnancies are truncated to just 3. If the total amount of pregnancies and outcomes matched, we consider the pregnancies in the last 12 months to be complete. Otherwise, 1 of the pregnancy is still incomplete.

### Ethical considerations

Informed consent was obtained from each head of the household and woman of reproductive age surveyed. A comprehensive understanding and agreement to participate was confirmed by signature or fingerprint of all participants prior to data collection. Ethical approval for the census was obtained from the Institutional Ethics Review Board for Health at CISM (CIBS-CISM) and from the CLIP co-ordinating centre at the University of British Columbia, prior to data collection.

## Results

The baseline census identified 50,493 inhabited households with 80,483 women of reproductive age (12 to 49 years). In relation to housing condition, there was a broad variation in materials used in construction: 37.1% (18,742) were constructed with conventional wall materials (cement blocks and burnt bricks) while 56.6% (28,586) were built with traditional materials (reed, bamboo, straw, wood, empty bags, paper bags and plastics). In terms of flooring material, 74.3% of households reported having conventional flooring material (timbre or parquet, marble, granite, mosaic and cement) while 25.2% (12,743) reported having compacted sand as the primary flooring material. Further details of the household construction materials can be found in S1 Table. The socio-economic status (SES) of households was generated using the principal component analysis method, this showed that SES was heterogeneous. In some regions 10.0% - 29.7% of households were in the poorest quintile while 3.9% - 32.7% of households were in the least poor quintile (Table 1).The mean age of WRA in this census was 26.9 years (SD± 10.13) and this varied by region from 26.3 and 27.4 years (Table 2). The clusters varied in the total number of households (from 1,317–5,914 households), number of women of reproductive age (1,962–9,553), and the average number of women per household (1.5–1.7) (Table 2).

**Table 1:** Poverty index of all households in the 12 clusters in Maputo and Gaza provinces in 2014.

Socio Economic Status (SES)	poorest	poorer	poor	less poor	least poor	Unknown
Maluana & Maciana	720	753	734	637	680	27
	20,3%	21,2%	20,7%	17,9%	19,1%	0,8%
Ilha Josina & Calanga	653	264	203	120	51	26
	49,6%	20,0%	15,4%	9,1%	3,9%	2,0%
3 de Fevereiro	1040	1103	1069	1083	1552	33
	17,7%	18,8%	18,2%	18,4%	26,4%	0,6%
Magude	1139	782	903	983	1182	495
	20,8%	14,3%	16,5%	17,9%	21,6%	9,0%
Messano	505	508	491	499	401	43
	20,6%	20,8%	20,1%	20,4%	16,4%	1,8%
Chaimite	1261	768	773	709	449	289
	29,7%	18,1%	18,2%	16,7%	10,6%	6,8%
Chissano	798	911	933	879	488	27
	19,8%	22,6%	23,1%	21,8%	12,1%	0,7%
Mazivila	712	762	780	615	298	104
	21,8%	23,3%	23,8%	18,8%	9,1%	3,2%
Chicumbane	435	851	715	919	1426	9
	10,0%	19,5%	16,4%	21,1%	32,7%	0,2%
Xilembene	1482	927	1095	1180	1096	102
	25,2%	15,8%	18,6%	20,1%	18,6%	1,7%
Chongoene	641	1153	1144	1344	1619	13
	10,8%	19,5%	19,3%	22,7%	27,4%	0,2%
Malehice	571	985	1022	895	619	15
	13,9%	24,0%	24,9%	21,8%	15,1%	0,4%
**Total**	**9957**	**9767**	**9862**	**9863**	**9861**	**1183**
	**19,7%**	**19,3%**	**19,5%**	**19,5%**	**19,5%**	**2,3%**

**Table 2:** Demographic information of women of reproductive age in in the 12 clusters of Gaza and Maputo province in 2014.

Name of Cluster	Households surveyed (N)	Total WRA	Age of WRA surveyed (mean +/- SD)	The average # of WRA per household (mean +/- SD)
Maluana+Maciana	3551	5302	27.0(10.12)	1.5(0.80)
Ilha Josina+Calanga	1317	1962	28.1(10.69)	1.5(0.82)
3 de Fevereiro	5880	9541	26.9(10.26)	1.6(0.90)
Magude	5484	8914	26.6(9.99)	1.6(0.93)
Messano	2447	3857	27.0(10.15)	1.6(0.88)
Chaimite	4249	6611	27.2(10.20)	1.6(0.84)
Chissano	4036	6435	26.9(10.11)	1.6(0.86)
Mazivila	3271	4862	27.4(10.08)	1.5(0.80)
Chicumbane	4355	7424	26.3(9.98)	1.7(0.97)
Xilembene	5882	9382	27.1(10.14)	1.6(0.85)
Chongoene	5914	9553	26.5(10.07)	1.6(0.85)
Malehice	4107	6640	26.9(10.15)	1.6(0.92)
** Total**	**50493**	**80483**	**26.9(10.13)**	**1.6(0.88)**

**Fig 2:**
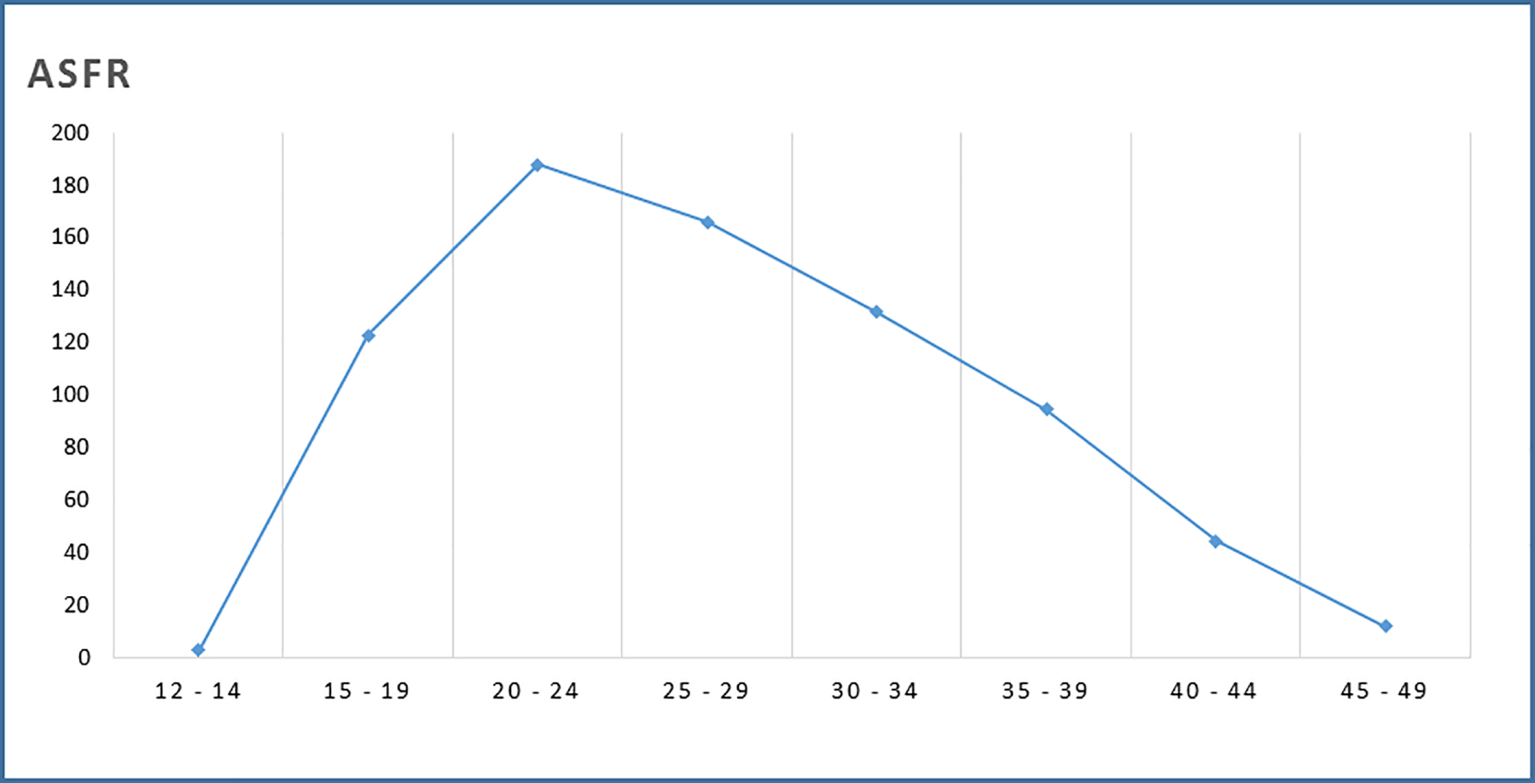
Age specific fertility rate in 12 clusters of Maputo and Gaza provinces in 2014.

The majority of women were married or engaged (47.4%) while single woman and separated/divorced/widowed women represented 44.0% and 8.2%, respectively. Agriculture was the main industry for employment, this was the field of employment for 43.3% of women of reproductive age in the labour force. A detailed breakdown of occupations are provided in S2 Table. In relation to the level of education, the baseline census indicated that only 55.8% of WRA achieved primary education, while 20.7% were illiterate. In terms of cluster distribution, illiteracy rates ranged from 12.3% to 33.3%. There was large variation in the percentage of secondary (1.7% - 22.8%) and higher level (0.0% - 1.1%) education (Table 3). Fig 2 shows the age specific fertility among the WRA in the 12 clusters. It is clear in this graph that the highest fertility occurs at 20–24 years; 188 live births per 1,000 women 20–24 years.

**Table 3:** The level of education of women of reproductive age in 12 clusters in Maputo and Gaza provinces in 2014.

Name of Cluster	Illiterate (n (%))	Primary school complete (n (%))	Secondary school complete (n (%))	High school complete (n (%))	Other (n (%))
Maluana+Maciana	1008 (19.0)	3105 (58.6)	581 (11.0)	8 (0.2)	596(11.2)
Ilha Josina+Calanga	6539 (33.3)	1007 (51.4)	34 (1.7)	6 (0.3)	261 (13.3)
3 de Fevereiro	2465 (25.9)	5448 (57.2)	890 (9.3)	13 (0.1)	716 (7.5)
Magude	1834 (20.7)	5032 (56.7)	1262 (14.2)	24 (0.3)	723 (8.1)
Messano	959 (25.0)	2143 (55.8)	304 (7.9)	6 (0.2)	428 (11.1)
Chaimite	1827 (27.7)	3362 (51.0)	236 (3.6)	1 (0.0)	1160 (17.6)
Chissano	1095 (17.1)	3010 (46.9)	548 (8.5)	6 (0.1)	1758 (27.4)
Mazivila	1472 (30.4)	2590 (53.5)	407 (8.4)	4 (0.1)	369 (7.6)
Chicumbane	991 (13.4)	4006 (54.1)	1162 (15.7)	27 (0.4)	1219 (16.5)
Xilembene	1854 (19.8)	5556 (59.5)	1195 (12.8)	9 (0.1)	728 (7.8)
Chongoene	1175 (12.3)	5435 (57.1)	2166 (22.8)	103 (1.1)	639 (6.7)
Malehice	1288 (19.5)	4039 (61.0)	962 (14.5)	4 (0.1)	328 (5.0)
**Total**	**16621 (20.7)**	**44733 (55.8)**	**9747 (12.1)**	**211 (0.3)**	**8925 (11.1)**

A total of 14,617 women were reported to have one or more pregnancies in the twelve months prior to the census, resulting in 9,029 (61.6%) completed pregnancies and 5,612 (38.4%) ongoing pregnancies during the interview. Of all completed pregnancies, 92.1% (8,796) resulted in live births, 4.9% (466) resulted in stillbirths and 3.0% (288) resulted in miscarriages. These data varied moderately across study clusters (Table 4).

**Table 4:** Fertility information of women of reproductive age in the 12 months prior to interview in Gaza and Maputo provinces in 2014.

Name of Cluster	Total number of women that reported one or more pregnancies (n (%))	Number of pregnancies resulting in a live birth (n (%))	Number of pregnancies resulting in a stillbirth (n (%))	Number of pregnancies resulting in a miscarriage (n (%))
Maluana+Maciana	1017 (7.0%)	627 (7.1%)	31 (6.7%)	18 (6.3%)
Ilha Josina+Calanga	428 (2.9%)	245 (2.8%)	18 (3.9%)	10 (3.5%)
3 de Fevereiro	1593 (10.9%)	1011 (11.5%)	46 (9.9%)	32 (11.1%)
Magude	1801(12.3%)	1080 (12.3%)	43 (9.2%)	24 (8.3%)
Messano	792 (5.4%)	489 (5.6%)	18 (3.9%)	13 (4.5%)
Chaimite	1490 (10.2%)	922 (10.5%)	32 (6.9%)	18 (6.3%)
Chissano	1036 (7.1%)	586 (6.7%)	46 (9.9%)	28 (9.7%)
Mazivila	937 (6.4%)	551 (6.3%)	43 (9.2%)	40 (13.9%)
Chicumbane	1298 (8.9%)	775 (8.8%)	37 (7.9%)	27 (9.4%)
Xilembene	1728 (11.8%)	1136 (12.9%)	79 (17.0%)	50 (17.4%)
Chongoene	1455 (10.0%)	773 (8.8%)	44 (9.4%)	14 (4.9%)
Malehice	1042 (7.1%)	601 (6.8%)	29 (6.2%)	14 (4.9%)
** Total**	**14617 (100.0)**	**8796 (100.0)**	**466 (100%)**	**288(100%)**

**Fig 3:**
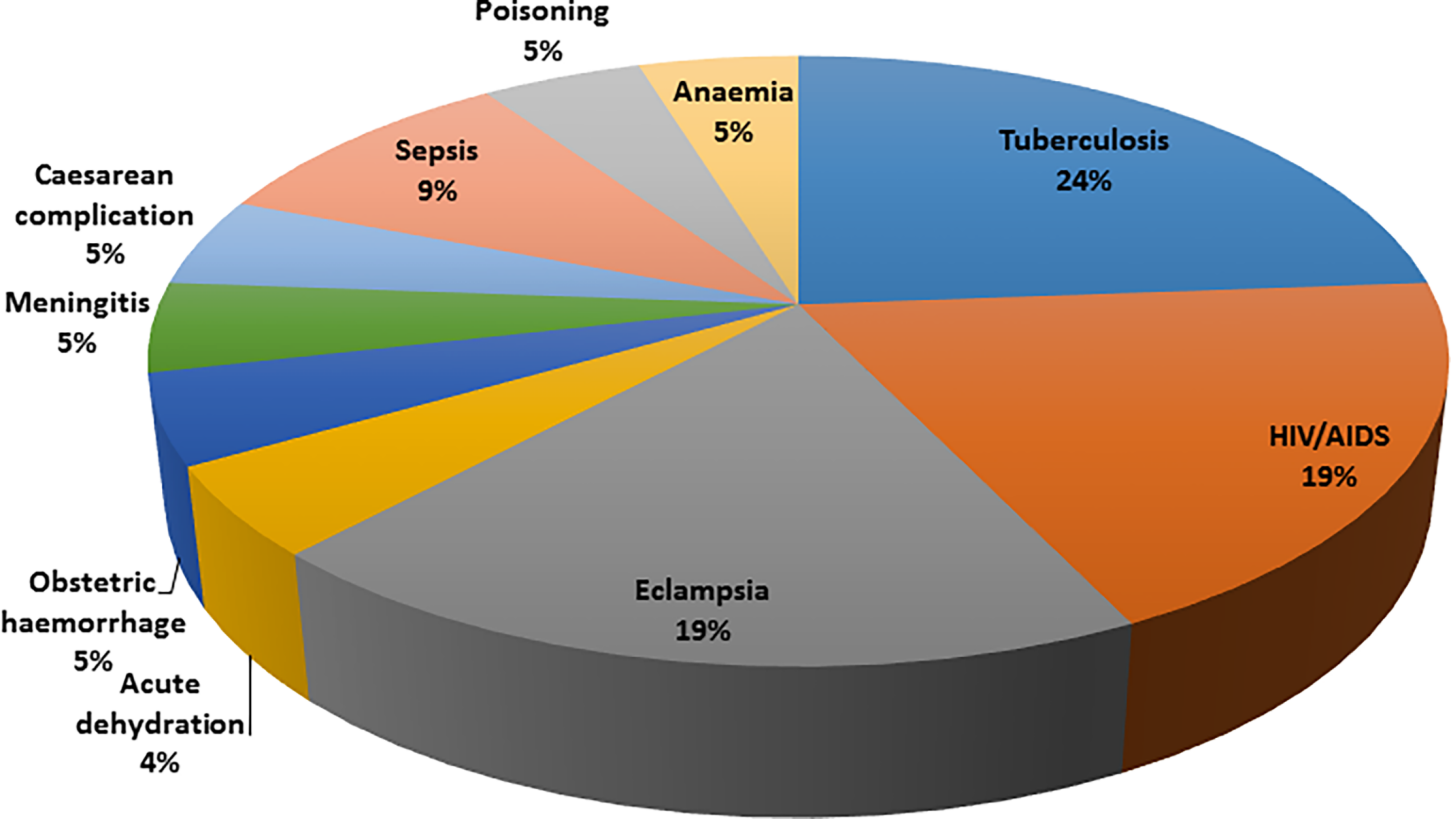
Causes of maternal death in 12 clusters of Maputo and Gaza provinces in 2014.

The baseline census identified 246 deaths of women of reproductive age. The death rate of WRA was 3.1 per 1,000 WRA. Among the death of women of reproductive age, 18 cases occurred during pregnancy or within 42 days of delivery or pregnancy termination, which resulted in a MMR of 204.6 per 100,000 live births. These results indicated that 61.1% of deaths occurred at health facility, 27.8% at home and 11.1% occurred somewhere else (for example on way to a health facility). All 18 maternal deaths were reviewed by physicians, including the findings of verbal autopsies. Twenty-one different diagnoses were found to be responsible for these deaths. Tuberculosis, HIV/AIDS and eclampsia are the primary causes of maternal deaths in these rural communities (Fig 3).

**Fig 4:**
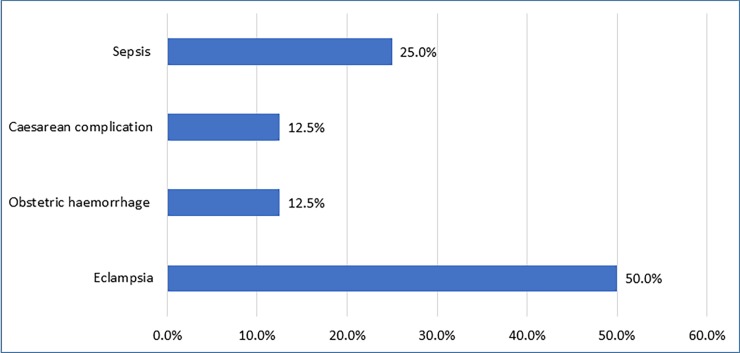
The direct obstetric causes of maternal death in 12 clusters of Maputo and Gaza provinces in 2014.

**Fig 5:**
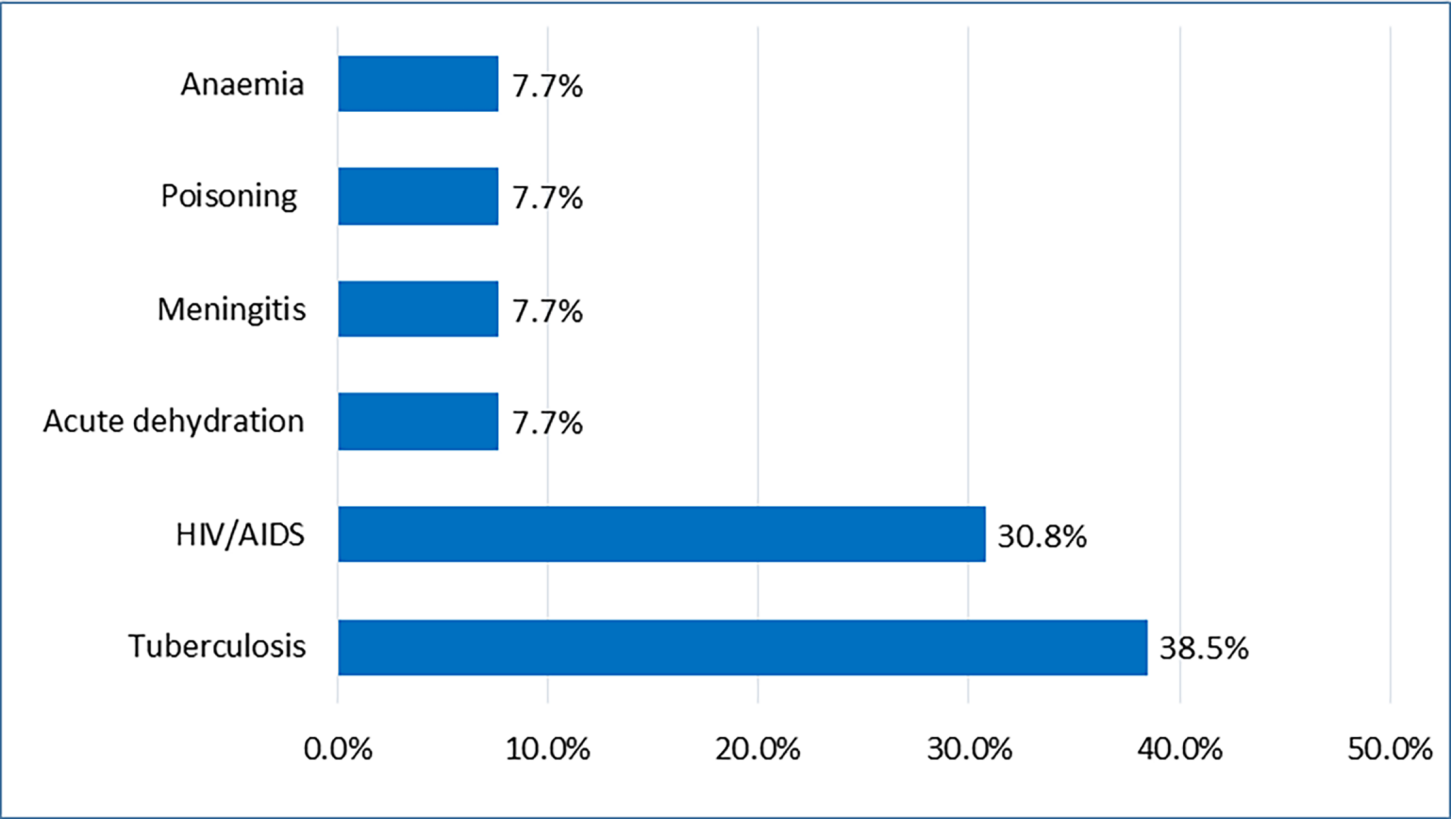
The indirect obstetric causes of maternal death in 12 clusters of Maputo and Gaza provinces in 2014.

The data showed that direct obstetric causes accounted for 38% of maternal deaths while indirect obstetric causes accounted for 62% of deaths. Eclampsia (50%) and sepsis (25%) were the most common direct obstetric causes of death while tuberculosis (38.5%) and HIV/AIDS (30.8%) were the major indirect causes (Figs 4 and 5). There was a total of 117 neonatal deaths reported during the census resulting in a NMR of 13.3 per 1,000 live births. In terms of cluster distribution, the neonatal mortality rate varied from 8.2 to 19.4 per 1,000 live births.

Table 5 compares the neonatal mortality rate between the Mozambique DHS 2011 and data reported in this study. It is clear that for overall neonatal mortality rate, as well as the neonatal mortality by place of residence, age and education were lower in this study as compared to the DHS. It is also important to see that the patterns of distribution among the age categories seems to be relatively different. In the DHS survey the high mortality rate is concentrated in the last age groups while in this study the highest rate occurs in the youngest age group. Mothers with primary school education are those with highest neonatal mortality rate.

**Table 5:** Neonatal mortality rates by area of residence, mother’s age and level of education in the 12 clusters from Gaza and Maputo provinces in 2014.

Characteristic	DHS 2011 (95% CI)	Study census 2014
Geographic Area		
Mozambique	31.0 (26.9–35.6)	-
Gaza and Maputo Provinces	35.2 (27.5–44.8)	-
Urban	36.7 (25.9–51.8)	-
Rural	33.7 (23.8–47.6)	13.3
Age categories (Gaza and Maputo Province)		
<20	37.4 (24.1–57.7)	16.9
20–29	29.5 (21.4–40.5)	12.0
30–39	42.2 (28.9–61.0)	12.2
40–49	70.8 (24.5–188.1)	15.1
Education		
None	33.3 (28.5–39.0)	11.7
Primary	33.0 (28.9–37.7)	14.2
Secondary	26.3 (19.0–36.3)	12.8

**Fig 6:**
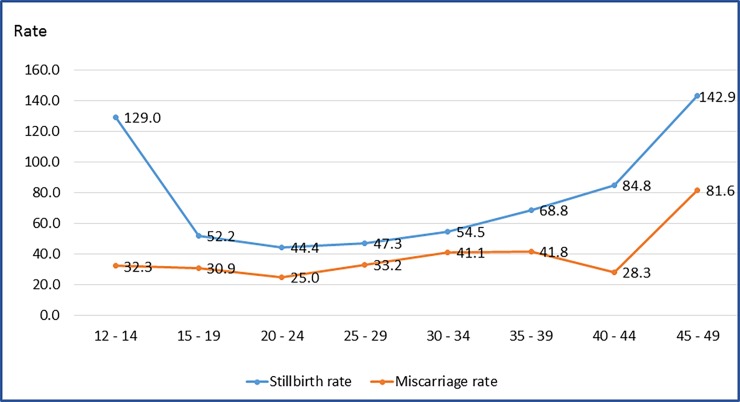
Age distribution of stillbirth rate and miscarriage rate in 12 clusters of Maputo and Gaza provinces in 2014.

The overall stillbirth rate in this study was 50.3/1,000 live birth. In terms of stillbirth rate distribution among clusters the results showed a variation from 33.5 per 1,000 live birth in Chaimite to 72.8 per 1,000 live birth in Chissano (Table 6). In terms of age specific distribution of stillbirth and miscarriage it can be seen in the Fig 6 that the stillbirth rate was higher in the first age group (12–14) and last age group (45–49). The rate of miscarriage, the graphs show that rate of miscarriage decreased from age group 12–14 (32.3 per 1,000 live birth) to age group 20–24 years old. Then the rates of miscarriage worsened from age 25–29 years old to the age 35–39 years old. The highest miscarriage rate was observed at age 45–49 years old.

**Table 6:** Mortality rates in the 12 clusters of Maputo and Gaza provinces in 2014.

Name of Cluster	Death Rate of WRA	Stillbirth Rate	Neonatal Mortality Rate
Maluana+Maciana	0.8	47.1	11.2
Ilha Josina+Calanga	0.5	68.4	8.2
3 de Fevereiro	0.9	43.5	11.9
Magude	4.4	38.3	14.8
Messano	4.4	35.5	18.4
Chaimite	3.8	33.5	8.7
Chissano	2.8	72.8	11.9
Mazivila	4.1	72.4	10.9
Chicumbane	2.6	45.6	10.3
Xilembene	4.3	65.0	19.4
Chongoene	3.7	53.9	19.4
Malehice	2.9	46.0	8.3
**Total**	**3.1**	**50.3**	**13.3**

## Discussion

This community-based census was conducted for all eligible households in the study areas of Maputo and Gaza provinces, Mozambique. Data were collected at the household and individual level. The data collected focused on demographic information, social and health indicators of women of reproductive age, as well as household characteristics and assets. This study highlights several challenges that the communities in rural Mozambique are facing. Findings indicate that access to adequate housing conditions is limited. Household assets varied within and between provinces when classified by poverty quintiles. Households in Gaza appeared to be in better condition than those in Maputo Province—this mirrors the findings of the National Health and Demographic Surveys conducted in 2011 [10].

There is variability in socio-economic status between clusters. The most dramatic difference was found in Ilha Josina Machel & Calanga where 49.6% of households were in the poorest quintile. Mozambique remains one of the poorest countries in the world with widespread inequalities. Most of the regions surveyed are located in rural areas where agriculture is the primary economic activity; however, agricultural methods are often outdated and weather-dependent, resulting in poor productivity [19, 20]. Ilha Josina & Calanga, Chaimite and Xilembene; the poorest clusters, are cyclically affected by floods which is likely to be the major contributor to high rates of poverty.

There was an average of 1.6 women of reproductive age per household. The low number of adult women per household could lead to challenges, particularly in regions with high rates of migration by men for employment. Few adults per household could impede access to health care while also caring for children. The fertility rate further highlights the need for ready access to health care services. This situation is similar in many African countries where the fertility rate is high and access to care and safe maternal health is limited [3].

About forty-seven percent of women of reproductive age were married or cohabitating at the time of survey. Some variation between clusters can be observed, for example in Ilha Josina & Calanga 58.0% of these women were married or cohabiting. Early marriage in rural areas has been associated with low levels of education in Mozambique [21, 22]. It is interesting to note that the clusters with higher proportion of education also reported a lower number of early marriages. The time spent in school, and a higher level of education is often associated with empowerment, which may explain this finding [23, 24].

The age specific fertility rate found in this census shows a similar pattern as that observed in 2011 in Mozambique during the DHS where fertility was higher in the age groups 15–19 and 20–24. Similarly with this census, the peaking of fertility in the DHS occurred at age 20–24 years. Conversely, it’s important to emphasize the age specific fertility rates in the 2011 DHS in Mozambique was higher than this census[10]. Contrarily, reports from South Africa and Tanzania in 2015 indicate the peak fertility rate occurs at age 25–29 years[25, 26].

Adverse pregnancy outcomes (stillbirth and miscarriage) as revealed in this study is in agreement with the findings of some analysis conducted in several places where advanced maternal age (>35 years of age) was at higher rate of perinatal death [27, 28], although the study revealed that the young adolescents (12–14 years) have also high rates of stillbirth.

The death rate among women of reproductive age was found to be 2.8 per 1,000. This finding is lower than that reported in the DHS in 2011 in Mozambique where the rate was 5.7 per 1,000 [10]. This may reflect a reduction in mortality that has recently occurred in Mozambique, even in areas where HIV and tuberculosis rates are high [29]. This study found a reduced maternal mortality rate in these communities as compared to the national estimates [10]. Socio-geographic disparities, especially in health systems between the south-centre and north of the country may be responsible for this rate difference. Provinces in the south typically have better socio-geographic conditions with better access to the health facilities than the centre and north regions that have a less robust health system [30]. In relation to the place of death, the results of this study are contrary to findings from the post-census mortality survey from 2007–2008 in Mozambique [31] where 61% of the maternal mortality occurred at home.

The overall neonatal mortality rate of 13.3 in this census is lower than national estimates published by the Ministry of Health for Maputo and Gaza Provinces where the neonatal mortality rate was 30.7 and 36.7 respectively [32]. Nevertheless, the rate is high in some clusters, which may be associated to the lack of improvements in the health system [30]. According to the mothers age, the patterns of neonatal mortality identified in this study is in agreement with 2011 DHS in Mozambique where rates are higher in both cases of early and advanced maternal age[10].

Although Ilha Josina Machel & Calanga had the lowest SES, they also had lower rates of mortality. This may indicate that there are community specific characteristics that have allowed them overcome the challenges related to low SES. Future studies should focus on identifying those characteristics associated with positive health outcomes to be replicated in other communities.

Tuberculosis, HIV/AIDS and eclampsia were the top three causes of maternal deaths in these communities. The indirect obstetric causes are similar to those from several studies in Africa, and in Mozambique, where the burden tuberculosis and HIV/AIDS (usually associated) on maternal mortality was high [9, 33, 34]. The impact of eclampsia in this study is inconsistent with other studies in Mozambique where obstetric haemorrhage was the most common direct cause [9]. Due to the low numbers of maternal deaths (18) the data on causes of maternal mortality should be interpreted with caution.

This study has several limitations. First, this is a cross-sectional analysis and therefore we cannot draw causal inferences from the results. Another limitation of this study is that the data were self-reported. The population was shown to be variably literate and may have had difficulty understanding the complex medical questions of this survey. Although the interviewers were carefully trained and supervised, some level of misclassification cannot be ruled out. There was also a potential for recall bias for fertility, mortality and during the administration of verbal autopsy forms. Causes of maternal death were based on verbal autopsies reviewed by physicians, this method also has inherent limitations [14, 35].

## Conclusion

This study is the first successful community-based data analysis providing socio-demographic and health estimates among women of reproductive age in Gaza and Maputo Province. The results of this study (population composition, household condition, fertility and mortality) showed marked differences between provinces and regions, indicating inequities in the region. Thus, represents the challenges and the needs for better and more decentralized health care services and programmes to guarantee safe reproductive and maternal health. This study revealed the potential of this demographic platform to estimate population level mortality ratios that can be used for the planning of health interventions. Continuous efforts and community-level initiatives should be maximized in order to improve the socio-economic and demographic conditions of rural women in Gaza and Maputo, as well as in Mozambique in general. The majority of births in these communities occurs at younger ages, especially in age group 20–24 years old indicating the need for sexual and reproductive services target to adolescents and youth. Pregnancies in women of advanced age (>35 years of age) were associated with higher rates of miscarriage and stillbirth. The high rate of miscarriage, and stillbirth in these rural communities emphasizes the urgent need for interventions to improve infant survival. Such interventions should address health, education, social and economic factors promoting the safe maternal and child health. The neonatal mortality rate in these communities was lower than the latest national estimates computed for both rural Maputo and Gaza provinces.

Tuberculosis and HIV/AIDS are prominent indirect causes of maternal death, while eclampsia represents the number one direct obstetric cause of maternal deaths for these communities. There is a need for implementation of proven interventions to improve maternal and child health in these communities.

## Supporting information

S1 Table Characteristics of households in the 12 clusters from Gaza and Maputo provinces in 2014. (DOCX)

S2 Table Reported marital status and occupation of women in the 12 clusters from Gaza and Maputo provinces in 2014. (PDF)

S3 Table Characteristics of households in study cluster areas. (PDF)

S4 Table Health experience of WRA during pregnancy. (PDF)

S5 Table Study dataset. (ZIP)
