# Supplementary material for: Health and socio-demographic profile of women of reproductive age in rural communities of southern Mozambique
Source: PLoS One. 2018 Feb 2;13(2):e0184249. doi: 10.1371/journal.pone.0184249 (PMC5796686; doi:10.1371/journal.pone.0184249)
Supplement: S1 Table — (DOCX) [file pone.0184249.s001.docx]

**S1 Table:** Characteristics of households in the 12 clusters from Gaza and Maputo Provinces in 2014

|  | Maluana & Maciana | | Ilha Josina& Calanga | | 3 de Fevereiro | | Magude | | Messano | | Chaimite | | Chissano | | Mazivila | | Chicumbane | | Xilembene | | Chongoene | | Malehice | | **Total** | |
| --- | --- | --- | --- | --- | --- | --- | --- | --- | --- | --- | --- | --- | --- | --- | --- | --- | --- | --- | --- | --- | --- | --- | --- | --- | --- | --- |
|  | **N** | **%** | **N** | **%** | **N** | **%** | **N** | **%** | **N** | **%** | **N** | **%** | **N** | **%** | **N** | **%** | **N** | **%** | **N** | **%** | **N** | **%** | **N** | **%** | **N** | **%** |
|  |  |  |  |  |  |  |  |  |  |  |  |  |  |  |  |  |  |  |  |  |  |  |  |  |  |  |
| Major flooring material in the house |  |  |  |  |  |  |  |  |  |  |  |  |  |  |  |  |  |  |  |  |  |  |  |  |  |  |
| Compacted with sand | 855 | 24.1% | 792 | 60.1% | 1490 | 25.3% | 1948 | 35.5% | 545 | 22.3% | 1572 | 37.0% | 905 | 22.4% | 812 | 24.8% | 390 | 9.0% | 2242 | 38.1% | 693 | 11.7% | 499 | 12.1% | 12743 | 25.2% |
| Timbre or parquet | 10 | 0.3% | 2 | 0.2% | 9 | 0.2% | 10 | 0.2% | 2 | 0.1% | 3 | 0.1% | 4 | 0.1% | 3 | 0.1% | 4 | 0.1% | 33 | 0.6% | 12 | 0.2% | 29 | 0.7% | 121 | 0.2% |
| Marble/ granite/ cement or mosaic | 2679 | 75.4% | 512 | 38.9% | 4359 | 74.1% | 3506 | 63.9% | 1892 | 77.3% | 2649 | 62.3% | 3115 | 77.2% | 2394 | 73.2% | 3954 | 90.8% | 3585 | 60.9% | 5202 | 88.0% | 3569 | 86.9% | 37416 | 74.1% |
| Unknown | 7 | 0.2% | 11 | 0.8% | 22 | 0.4% | 20 | 0.4% | 8 | 0.3% | 25 | 0.6% | 12 | 0.3% | 62 | 1.9% | 7 | 0.2% | 22 | 0.4% | 7 | 0.1% | 10 | 0.2% | 213 | 0.4% |
|  |  |  |  |  |  |  |  |  |  |  |  |  |  |  |  |  |  |  |  |  |  |  |  |  |  |  |
| Major wall material in house |  |  |  |  |  |  |  |  |  |  |  |  |  |  |  |  |  |  |  |  |  |  |  |  |  |  |
| Reed/ bamboo/ straw/ timbre or wood | 1659 | 46.7% | 990 | 75.2% | 2363 | 40.2% | 2151 | 39.2% | 1456 | 59.5% | 2486 | 58.5% | 2443 | 60.5% | 1913 | 58.5% | 2794 | 64.2% | 3435 | 58.4% | 4106 | 69.4% | 2612 | 63.6% | 28408 | 56.3% |
| Empty bags/ paper bags or plastics | 5 | 0.1% | 2 | 0.2% | 17 | 0.3% | 19 | 0.3% | 6 | 0.2% | 12 | 0.3% | 8 | 0.2% | 15 | 0.5% | 8 | 0.2% | 40 | 0.7% | 16 | 0.3% | 30 | 0.7% | 178 | 0.4% |
| Metal zincs | 7 | 0.2% | 3 | 0.2% | 51 | 0.9% | 119 | 2.2% | 27 | 1.1% | 214 | 5.0% | 240 | 5.9% | 80 | 2.4% | 83 | 1.9% | 53 | 0.9% | 25 | 0.4% | 25 | 0.6% | 927 | 1.8% |
| Clay bricks | 64 | 1.8% | 18 | 1.4% | 233 | 4.0% | 137 | 2.5% | 44 | 1.8% | 72 | 1.7% | 79 | 2.0% | 170 | 5.2% | 115 | 2.6% | 230 | 3.9% | 47 | 0.8% | 73 | 1.8% | 1282 | 2.5% |
| Cement blocks or burnt bricks | 1802 | 50.7% | 298 | 22.6% | 3205 | 54.5% | 2580 | 47.0% | 880 | 36.0% | 1197 | 28.2% | 1251 | 31.0% | 1048 | 32.0% | 1353 | 31.1% | 2050 | 34.9% | 1716 | 29.0% | 1362 | 33.2% | 18742 | 37.1% |
| Missing | 14 | 0.4% | 6 | 0.5% | 11 | 0.2% | 478 | 8.7% | 34 | 1.4% | 268 | 6.3% | 15 | 0.4% | 45 | 1.4% | 2 | 0.0% | 74 | 1.3% | 4 | 0.1% | 5 | 0.1% | 956 | 1.9% |
|  |  |  |  |  |  |  |  |  |  |  |  |  |  |  |  |  |  |  |  |  |  |  |  |  |  |  |
| Type of latrine used |  |  |  |  |  |  |  |  |  |  |  |  |  |  |  |  |  |  |  |  |  |  |  |  |  |  |
| Traditional (not improved) | 2595 | 73.1% | 693 | 52.6% | 4544 | 77.3% | 3124 | 57.0% | 1681 | 68.7% | 2393 | 56.3% | 3125 | 77.4% | 1635 | 50.0% | 3219 | 73.9% | 4075 | 69.3% | 4178 | 70.6% | 3258 | 79.3% | 34520 | 68.4% |
| Traditional (improved) | 302 | 8.5% | 31 | 2.4% | 789 | 13.4% | 647 | 11.8% | 209 | 8.5% | 305 | 7.2% | 636 | 15.8% | 325 | 9.9% | 821 | 18.9% | 863 | 14.7% | 1322 | 22.4% | 722 | 17.6% | 6972 | 13.8% |
| Improved conventional | 172 | 4.8% | 24 | 1.8% | 270 | 4.6% | 218 | 4.0% | 102 | 4.2% | 179 | 4.2% | 138 | 3.4% | 191 | 5.8% | 122 | 2.8% | 179 | 3.0% | 218 | 3.7% | 97 | 2.4% | 1910 | 3.8% |
| Retreat connected by septic tank | 116 | 3.3% | 8 | 0.6% | 172 | 2.9% | 133 | 2.4% | 39 | 1.6% | 28 | 0.7% | 18 | 0.4% | 16 | 0.5% | 93 | 2.1% | 20 | 0.3% | 131 | 2.2% | 13 | 0.3% | 787 | 1.6% |
| No latrine | 361 | 10.2% | 549 | 41.7% | 105 | 1.8% | 1362 | 24.8% | 415 | 17.0% | 1337 | 31.5% | 119 | 2.9% | 1101 | 33.7% | 99 | 2.3% | 744 | 12.6% | 62 | 1.0% | 16 | 0.4% | 6270 | 12.4% |
| Missing | 5 | 0.1% | 12 | 0.9% | 0 | 0.0% | 0 | 0.0% | 1 | 0.0% | 7 | 0.2% | 0 | 0.0% | 3 | 0.1% | 1 | 0.0% | 1 | 0.0% | 3 | 0.1% | 1 | 0.0% | 34 | 0.1% |
|  |  |  |  |  |  |  |  |  |  |  |  |  |  |  |  |  |  |  |  |  |  |  |  |  |  |  |
| Main source of power for illumination |  |  |  |  |  |  |  |  |  |  |  |  |  |  |  |  |  |  |  |  |  |  |  |  |  |  |
| Firewood | 20 | 0.6% | 8 | 0.6% | 96 | 1.6% | 20 | 0.4% | 3 | 0.1% | 20 | 0.5% | 139 | 3.4% | 42 | 1.3% | 45 | 1.0% | 66 | 1.1% | 102 | 1.7% | 64 | 1.6% | 625 | 1.2% |
| Batteries | 100 | 2.8% | 17 | 1.3% | 25 | 0.4% | 137 | 2.5% | 42 | 1.7% | 132 | 3.1% | 51 | 1.3% | 47 | 1.4% | 9 | 0.2% | 41 | 0.7% | 71 | 1.2% | 56 | 1.4% | 728 | 1.4% |
| Petroleum lamp or candles | 2186 | 61.6% | 1153 | 87.5% | 3243 | 55.2% | 3094 | 56.4% | 1485 | 60.7% | 2773 | 65.3% | 3134 | 77.7% | 2612 | 79.9% | 1917 | 44.0% | 2825 | 48.0% | 2637 | 44.6% | 2930 | 71.3% | 29989 | 59.4% |
| Public electricity | 1120 | 31.5% | 97 | 7.4% | 2480 | 42.2% | 2086 | 38.0% | 870 | 35.6% | 1146 | 27.0% | 601 | 14.9% | 338 | 10.3% | 2343 | 53.8% | 2778 | 47.2% | 2781 | 47.0% | 849 | 20.7% | 17489 | 34.6% |
| Generators or solar panel | 79 | 2.2% | 32 | 2.4% | 18 | 0.3% | 131 | 2.4% | 38 | 1.6% | 120 | 2.8% | 97 | 2.4% | 168 | 5.1% | 34 | 0.8% | 71 | 1.2% | 86 | 1.5% | 125 | 3.0% | 999 | 2.0% |
| other | 45 | 1.3% | 10 | 0.8% | 17 | 0.3% | 14 | 0.3% | 9 | 0.4% | 57 | 1.3% | 14 | 0.3% | 62 | 1.9% | 7 | 0.2% | 96 | 1.6% | 237 | 4.0% | 80 | 1.9% | 648 | 1.3% |
| Missing | 1 | 0.0% | 0 | 0.0% | 1 | 0.0% | 2 | 0.0% | 0 | 0.0% | 1 | 0.0% | 0 | 0.0% | 2 | 0.1% | 0 | 0.0% | 5 | 0.1% | 0 | 0.0% | 3 | 0.1% | 15 | 0.0% |
|  |  |  |  |  |  |  |  |  |  |  |  |  |  |  |  |  |  |  |  |  |  |  |  |  |  |  |
|  |  |  |  |  |  |  |  |  |  |  |  |  |  |  |  |  |  |  |  |  |  |  |  |  |  |  |
| Household source of drinking water in the yard | 1225 | 34.5% | 101 | 7.7% | 2400 | 40.8% | 1196 | 21.8% | 412 | 16.8% | 97 | 2.3% | 773 | 19.2% | 390 | 11.9% | 2283 | 52.4% | 648 | 11.0% | 1287 | 21.8% | 710 | 17.3% | 11522 | 22.8% |
| Missing or unknown | 0 | 0.0% | 0 | 0.0% | 1 | 0.0% | 2 | 0.0% | 1 | 0.0% | 0 | 0.0% | 2 | 0.0% | 1 | 0.0% | 2 | 0.0% | 1 | 0.0% | 2 | 0.0% | 8 | 0.2% | 20 | 0.0% |
