# Supplementary material for: Health and socio-demographic profile of women of reproductive age in rural communities of southern Mozambique
Source: PLoS One. 2018 Feb 2;13(2):e0184249. doi: 10.1371/journal.pone.0184249 (PMC5796686; doi:10.1371/journal.pone.0184249)
Supplement: S2 Table — (PDF) [file pone.0184249.s002.pdf]

Supplementary Table 2: Reported occupation of women in study clusters in Maputo and Gaza

Occupational area of wom	Maluana & Maciana		Ilha Josina& Calanga		3 de Fevereiro		Magude		Messano		Chaimite	
	**N**	**%**	**N**	**%**	**N**	**%**	**N**	**%**	**N**	**%**	**N**	**%**
Agriculture	2054	38.80%	1082	55.20%	3440	36.10%	2982	33.60%	1919	50.00%	4503	68.40%
Education	880	16.60%	192	9.80%	1602	16.80%	1606	18.10%	683	17.80%	914	13.90%
Culture	3	0.10%	4	0.20%	14	0.10%	28	0.30%	2	0.10%	5	0.10%
Finance/ Banking	86	1.60%	15	0.80%	158	1.70%	122	1.40%	57	1.50%	38	0.60%
Communication and transport	1	0.00%	0	0.00%	2	0.00%	2	0.00%	0	0.00%	0	0.00%
Tourism	2	0.00%	0	0.00%	0	0.00%	9	0.10%	4	0.10%	2	0.00%
Security or army	4	0.10%	1	0.10%	2	0.00%	8	0.10%	1	0.00%	0	0.00%
Other public service	86	1.60%	12	0.60%	134	1.40%	209	2.40%	24	0.60%	42	0.60%
Informal sector	145	2.70%	22	1.10%	221	2.30%	325	3.70%	96	2.50%	89	1.40%
Industry	38	0.70%	2	0.10%	50	0.50%	86	1.00%	5	0.10%	2	0.00%
Does not work	1989	37.50%	629	32.10%	3898	40.90%	3481	39.20%	1040	27.10%	987	15.00%
Unknown	10	0.20%	2	0.10%	11	0.10%	17	0.20%	9	0.20%	4	0.10%

Chissano		Mazivila		Chicumbane		Xilembene		Chongoene		Malehice		Total	
**N**	**%**	**N**	**%**	**N**	**%**	**N**	**%**	**N**	**%**	**N**	**%**	**N**	**%**
1399	21.80%	3696	76.30%	2997	40.50%	3915	41.90%	3436	36.10%	3300	49.80%	34723	43.30%
538	8.40%	739	15.30%	1146	15.50%	1221	13.10%	2418	25.40%	1012	15.30%	12951	16.10%
1	0.00%	3	0.10%	5	0.10%	4	0.00%	6	0.10%	2	0.00%	77	0.10%
17	0.30%	29	0.60%	95	1.30%	64	0.70%	93	1.00%	12	0.20%	786	1.00%
1	0.00%	2	0.00%	0	0.00%	2	0.00%	7	0.10%	1	0.00%	18	0.00%
4	0.10%	6	0.10%	3	0.00%	4	0.00%	17	0.20%	4	0.10%	55	0.10%
0	0.00%	0	0.00%	0	0.00%	3	0.00%	8	0.10%	2	0.00%	29	0.00%
43	0.70%	30	0.60%	184	2.50%	91	1.00%	176	1.80%	40	0.60%	1071	1.30%
30	0.50%	3	0.10%	177	2.40%	74	0.80%	211	2.20%	31	0.50%	1424	1.80%
3	0.00%	0	0.00%	9	0.10%	6	0.10%	14	0.10%	3	0.00%	218	0.30%
4371	68.10%	329	6.80%	2785	37.60%	3949	42.30%	3115	32.70%	2203	33.30%	28776	35.90%
10	0.20%	5	0.10%	4	0.10%	9	0.10%	17	0.20%	11	0.20%	109	0.10%
