# Supplementary material for: Health and socio-demographic profile of women of reproductive age in rural communities of southern Mozambique
Source: PLoS One. 2018 Feb 2;13(2):e0184249. doi: 10.1371/journal.pone.0184249 (PMC5796686; doi:10.1371/journal.pone.0184249)
Supplement: S3 Table — (PDF) [file pone.0184249.s003.pdf]

	Maluana & Maciana		Ilha Josina& Calanga		3 de Fevereiro		Magude		Messano		Chaimite		Chissano		Mazivila		Chicumbane		Xilembene		Chongoene		Malehice		Total	
	N	%	N	%	N	%	N	%	N	%	N	%	N	%	N	%	N	%	N	%	N	%	N	%	N	%
Number of households with one or more married women of reproductive age 12-49 (WRA)	3551		1317		5880		5484		2447		4249		4036		3271		4355		5882		5914		4107		50493	
Number of people living in a household	18536		7499		34445		31452		13400		25601		23647		20342		26410		36663		32760		23554		294309	
Number of people living in a household (mean (sd))	5.2 (2.50)		5.7 (2.95)		5.9 (2.83)		5.7 (3.10)		5.5 (2.80)		6.0 (3.07)		5.9 (2.93)		6.2 (3.27)		6.1 (3.04)		6.2 (3.04)		5.5 (2.68)		5.7 (2.97)		5.8 (2.95)	
Major flooring material in the house																										
Compacted with sand	855	24.10%	792	60.10%	1490	25.30%	1948	35.50%	545	22.30%	1572	37.00%	905	22.40%	812	24.80%	390	9.00%	2242	38.10%	693	11.70%	499	12.10%	12743	25.20%
Timbre or parquet	10	0.30%	2	0.20%	9	0.20%	10	0.20%	2	0.10%	3	0.10%	4	0.10%	3	0.10%	4	0.10%	33	0.60%	12	0.20%	29	0.70%	121	0.20%
Marble/ granite/ cement or mosaic	2679	75.40%	512	38.90%	4359	74.10%	3506	63.90%	1892	77.30%	2649	62.30%	3115	77.20%	2394	73.20%	3954	90.80%	3585	60.90%	5202	88.00%	3569	86.90%	37416	74.10%
unknown	7	0.20%	11	0.80%	22	0.40%	20	0.40%	8	0.30%	25	0.60%	12	0.30%	62	1.90%	7	0.20%	22	0.40%	7	0.10%	10	0.20%	213	0.40%
Major wall material in house																										
Reed/ bamboo/ straw/ timbre or wood	1659	46.70%	990	75.20%	2363	40.20%	2151	39.20%	1456	59.50%	2486	58.50%	2443	60.50%	1913	58.50%	2794	64.20%	3435	58.40%	4106	69.40%	2612	63.60%	28408	56.30%
Empty bags/ paper bags or plastics	5	0.10%	2	0.20%	17	0.30%	19	0.30%	6	0.20%	12	0.30%	8	0.20%	15	0.50%	8	0.20%	40	0.70%	16	0.30%	30	0.70%	178	0.40%
Metal zincs	7	0.20%	3	0.20%	51	0.90%	119	2.20%	27	1.10%	214	5.00%	240	5.90%	80	2.40%	83	1.90%	53	0.90%	25	0.40%	25	0.60%	927	1.80%
Clay bricks	64	1.80%	18	1.40%	233	4.00%	137	2.50%	44	1.80%	72	1.70%	79	2.00%	170	5.20%	115	2.60%	230	3.90%	47	0.80%	73	1.80%	1282	2.50%
Cement blocks or burnt bricks	1802	50.70%	298	22.60%	3205	54.50%	2580	47.00%	880	36.00%	1197	28.20%	1251	31.00%	1048	32.00%	1353	31.10%	2050	34.90%	1716	29.00%	1362	33.20%	18742	37.10%
Missing	14	0.40%	6	0.50%	11	0.20%	478	8.70%	34	1.40%	268	6.30%	15	0.40%	45	1.40%	2	0.00%	74	1.30%	4	0.10%	5	0.10%	956	1.90%
Type of latrine used																										
Traditional (not improved)	2595	73.10%	693	52.60%	4544	77.30%	3124	57.00%	1681	68.70%	2393	56.30%	3125	77.40%	1635	50.00%	3219	73.90%	4075	69.30%	4178	70.60%	3258	79.30%	34520	68.40%
Traditional (improved)	302	8.50%	31	2.40%	789	13.40%	647	11.80%	209	8.50%	305	7.20%	636	15.80%	325	9.90%	821	18.90%	863	14.70%	1322	22.40%	722	17.60%	6972	13.80%
Improved conventional	172	4.80%	24	1.80%	270	4.60%	218	4.00%	102	4.20%	179	4.20%	138	3.40%	191	5.80%	122	2.80%	179	3.00%	218	3.70%	97	2.40%	1910	3.80%
Retreat connected by septic tank	116	3.30%	8	0.60%	172	2.90%	133	2.40%	39	1.60%	28	0.70%	18	0.40%	16	0.50%	93	2.10%	20	0.30%	131	2.20%	13	0.30%	787	1.60%
No latrine	361	10.20%	549	41.70%	105	1.80%	1362	24.80%	415	17.00%	1337	31.50%	119	2.90%	1101	33.70%	99	2.30%	744	12.60%	62	1.00%	16	0.40%	6270	12.40%
Missing	5	0.10%	12	0.90%	0	0.00%	0	0.00%	1	0.00%	7	0.20%	0	0.00%	3	0.10%	1	0.00%	1	0.00%	3	0.10%	1	0.00%	34	0.10%
Main source of power for illumination																										
Firewood	20	0.60%	8	0.60%	96	1.60%	20	0.40%	3	0.10%	20	0.50%	139	3.40%	42	1.30%	45	1.00%	66	1.10%	102	1.70%	64	1.60%	625	1.20%
Batteries	100	2.80%	17	1.30%	25	0.40%	137	2.50%	42	1.70%	132	3.10%	51	1.30%	47	1.40%	9	0.20%	41	0.70%	71	1.20%	56	1.40%	728	1.40%
Petroleum lamp or candles	2186	61.60%	1153	87.50%	3243	55.20%	3094	56.40%	1485	60.70%	2773	65.30%	3134	77.70%	2612	79.90%	1917	44.00%	2825	48.00%	2637	44.60%	2930	71.30%	29989	59.40%
Public electricity	1120	31.50%	97	7.40%	2480	42.20%	2086	38.00%	870	35.60%	1146	27.00%	601	14.90%	338	10.30%	2343	53.80%	2778	47.20%	2781	47.00%	849	20.70%	17489	34.60%
Generators or solar panel	79	2.20%	32	2.40%	18	0.30%	131	2.40%	38	1.60%	120	2.80%	97	2.40%	168	5.10%	34	0.80%	71	1.20%	86	1.50%	125	3.00%	999	2.00%
other	45	1.30%	10	0.80%	17	0.30%	14	0.30%	9	0.40%	57	1.30%	14	0.30%	62	1.90%	7	0.20%	96	1.60%	237	4.00%	80	1.90%	648	1.30%
Missing	1	0.00%	0	0.00%	1	0.00%	2	0.00%	0	0.00%	1	0.00%	0	0.00%	2	0.10%	0	0.00%	5	0.10%	0	0.00%	3	0.10%	15	0.00%
Household has an iron	1603	45.10%	325	24.70%	2454	41.70%	2419	44.10%	930	38.00%	1338	31.50%	1635	40.50%	1041	31.80%	2077	47.70%	1905	32.40%	3132	53.00%	1566	38.10%	20425	40.50%
Missing or unknown	0	0.00%	3	0.20%	4	0.10%	11	0.20%	3	0.10%	7	0.20%	15	0.40%	1	0.00%	4	0.10%	7	0.10%	72	1.20%	23	0.60%	150	0.30%
Household has one or more clock or watch	827	23.30%	221	16.80%	1168	19.90%	1103	20.10%	467	19.10%	748	17.60%	540	13.40%	512	15.70%	1175	27.00%	837	14.20%	1537	26.00%	540	13.10%	9675	19.20%
Missing or unknown	0	0.00%	0	0.00%	11	0.20%	13	0.20%	4	0.20%	3	0.10%	22	0.50%	3	0.10%	2	0.00%	11	0.20%	20	0.30%	6	0.10%	95	0.20%
Household has a radio of any sort	1878	52.90%	616	46.80%	2945	50.10%	3209	58.50%	1380	56.40%	2473	58.20%	2693	66.70%	1672	51.10%	2964	68.10%	3365	57.20%	3985	67.40%	2533	61.70%	29713	58.80%
Missing or unknown	1	0.00%	0	0.00%	4	0.10%	2	0.00%	0	0.00%	6	0.10%	5	0.10%	0	0.00%	4	0.10%	6	0.10%	6	0.10%	14	0.30%	48	0.10%
Type of transportation owned by household																										
None	2785	78.40%	928	70.50%	3952	67.20%	3325	60.60%	1713	70.00%	2666	62.70%	2881	71.40%	2634	80.50%	3240	74.40%	3817	64.90%	4375	74.00%	2959	72.00%	35275	69.90%
Bicycle	502	14.10%	275	20.90%	1451	24.70%	1518	27.70%	536	21.90%	1293	30.40%	818	20.30%	372	11.40%	599	13.80%	1357	23.10%	955	16.10%	828	20.20%	10504	20.80%
Motorbike	34	1.00%	3	0.20%	40	0.70%	190	3.50%	60	2.50%	63	1.50%	67	1.70%	51	1.60%	56	1.30%	197	3.30%	151	2.60%	60	1.50%	972	1.90%
Boat	2	0.10%	15	1.10%	6	0.10%	4	0.10%	12	0.50%	5	0.10%	3	0.10%	2	0.10%	27	0.60%	2	0.00%	4	0.10%	5	0.10%	87	0.20%
Car	136	3.80%	21	1.60%	307	5.20%	400	7.30%	98	4.00%	163	3.80%	252	6.20%	192	5.90%	284	6.50%	334	5.70%	415	7.00%	242	5.90%	2844	5.60%
Other	92	2.60%	72	5.50%	119	2.00%	45	0.80%	28	1.10%	58	1.40%	10	0.20%	17	0.50%	148	3.40%	171	2.90%	12	0.20%	8	0.20%	780	1.50%
Missing or unknown	0	0.00%	3	0.20%	5	0.10%	2	0.00%	0	0.00%	1	0.00%	5	0.10%	3	0.10%	1	0.00%	4	0.10%	2	0.00%	5	0.10%	31	0.10%
Number of beds in household																										
One	1643	46.30%	545	41.40%	2313	39.30%	2238	40.80%	1078	44.10%	1844	43.40%	1715	42.50%	1347	41.20%	1811	41.60%	2367	40.20%	2508	42.40%	1635	39.80%	21044	41.70%
Two or more	1132	31.90%	332	25.20%	2631	44.70%	2390	43.60%	937	38.30%	1542	36.30%	1808	44.80%	1344	41.10%	2000	45.90%	2690	45.70%	2644	44.70%	1955	47.60%	21405	42.40%
None	774	21.80%	439	33.30%	932	15.90%	854	15.60%	431	17.60%	859	20.20%	503	12.50%	579	17.70%	541	12.40%	822	14.00%	746	12.60%	500	12.20%	7980	15.80%
Missing or unknown	2	0.10%	1	0.10%	4	0.10%	2	0.00%	1	0.00%	4	0.10%	10	0.20%	1	0.00%	3	0.10%	3	0.10%	16	0.30%	17	0.40%	64	0.10%
Number of bedrooms in household																										
One	1182	33.30%	357	27.10%	1513	25.70%	1444	26.30%	736	30.10%	815	19.20%	1153	28.60%	666	20.40%	1171	26.90%	950	16.20%	1620	27.40%	943	23.00%	12550	24.90%
Two	1154	32.50%	362	27.50%	1929	32.80%	1619	29.50%	687	28.10%	1070	25.20%	906	22.40%	1053	32.20%	1386	31.80%	1407	23.90%	2182	36.90%	1053	25.60%	14808	29.30%
Three or more	830	23.40%	316	24.00%	1961	33.40%	1742	31.80%	622	25.40%	1113	26.20%	946	23.40%	1202	36.70%	1337	30.70%	1949	33.10%	1489	25.20%	1201	29.20%	14708	29.10%
No division of rooms	384	10.80%	282	21.40%	474	8.10%	674	12.30%	402	16.40%	1251	29.40%	1027	25.40%	350	10.70%	460	10.60%	1575	26.80%	616	10.40%	907	22.10%	8402	16.60%
Missing or unknown	1	0.00%	0	0.00%	3	0.10%	5	0.10%	0	0.00%	0	0.00%	4	0.10%	0	0.00%	1	0.00%	1	0.00%	7	0.10%	3	0.10%	25	0.00%
Household has a TV	1374	38.70%	270	20.50%	2601	44.20%	2444	44.60%	1031	42.10%	1488	35.00%	1796	44.50%	1262	38.60%	2415	55.50%	2798	47.60%	3275	55.40%	1836	44.70%	22590	44.70%
Missing or unknown	0	0.00%	0	0.00%	3	0.10%	1	0.00%	0	0.00%	0	0.00%	7	0.20%	1	0.00%	3	0.10%	6	0.10%	6	0.10%	7	0.20%	34	0.10%
Household has a phone																										
Ground line	1	0.00%	0	0.00%	1	0.00%	11	0.20%	0	0.00%	1	0.00%	1	0.00%	8	0.20%	4	0.10%	152	2.60%	11	0.20%	19	0.50%	209	0.40%
Cell phone	3301	93.00%	1136	86.30%	5461	92.90%	4972	90.70%	2211	90.40%	3913	92.10%	3787	93.80%	2996	91.60%	4127	94.80%	5121	87.10%	5522	93.40%	3840	93.50%	46387	91.90%
Both	65	1.80%	28	2.10%	60	1.00%	207	3.80%	92	3.80%	9	0.20%	1	0.00%	37	1.10%	16	0.40%	198	3.40%	162	2.70%	29	0.70%	904	1.80%
None	183	5.20%	153	11.60%	358	6.10%	294	5.40%	144	5.90%	325	7.60%	245	6.10%	230	7.00%	208	4.80%	409	7.00%	219	3.70%	218	5.30%	2986	5.90%
Missing or unknown	1	0.00%	0	0.00%	0	0.00%	0	0.00%	0	0.00%	1	0.00%	2	0.00%	0	0.00%	0	0.00%	2	0.00%	0	0.00%	1	0.00%	7	0.00%
Household source of drinking water in the yard	1225	34.50%	101	7.70%	2400	40.80%	1196	21.80%	412	16.80%	97	2.30%	773	19.20%	390	11.90%	2283	52.40%	648	11.00%	1287	21.80%	710	17.30%	11522	22.80%
Missing or unknown	0	0.00%	0	0.00%	1	0.00%	2	0.00%	1	0.00%	0	0.00%	2	0.00%	1	0.00%	2	0.00%	1	0.00%	2	0.00%	8	0.20%	20	0.00%
