# Supplementary material for: Health and socio-demographic profile of women of reproductive age in rural communities of southern Mozambique
Source: PLoS One. 2018 Feb 2;13(2):e0184249. doi: 10.1371/journal.pone.0184249 (PMC5796686; doi:10.1371/journal.pone.0184249)
Supplement: S4 Table — (PDF) [file pone.0184249.s004.pdf]

Supplementary Table 4: Health experience of WRA during pregnancy

	Maluana & Maciana		Ilha Josina& Calanga		3 de Fevereiro		Magude		Messano		Chaimite		Chissano		Mazivila		Chicumbane		Xilembene		Chongoene		Malehice		**Total**	
	N	%	N	%	N	%	N	%	N	%	N	%	N	%	N	%	N	%	N	%	N	%	N	%	N	%
Pregnancies complicated by high blood pressure	193	3,60%	67	3,40%	221	2,30%	412	4,60%	163	4,20%	369	5,60%	209	3,30%	177	3,70%	400	5,40%	622	6,70%	410	4,30%	364	5,50%	3607	4,50%
Women with history of convulsion	139	2,60%	73	3,70%	170	1,80%	526	5,90%	208	5,40%	510	7,70%	328	5,10%	125	2,60%	451	6,10%	487	5,20%	547	5,70%	350	5,30%	3914	4,90%
